# Patents and Innovation Among Neurosurgeons from the American Association of Neurological Surgeons

**DOI:** 10.7759/cureus.7031

**Published:** 2020-02-18

**Authors:** Rebecca B Baron, Remi A Kessler, Ansh Bhammar, Nicholas Boulis, John R. Adler, Karan Kohli, Constantinos Hadjipanayis

**Affiliations:** 1 Neurosurgery, Icahn School of Medicine at Mount Sinai, New York, USA; 2 Neurosurgery, Johns Hopkins University, Baltimore, USA; 3 Neurosurgery, Emory University School of Medicine, Atlanta, USA; 4 Radiation Oncology, Stanford University Medical Center, Stanford, USA; 5 Department of Neurosurgery, Stanford University School of Medicine, Stanford, CA, USA; 6 Neurosurgery, Icahn School at Mount Sinai, New York, USA

**Keywords:** innovation, medical devices, neurosurgery, patent, technology, uspto, aans

## Abstract

Objective

Neurosurgeons have taken on the role of innovators, continuing to move the field forward over the centuries. More recently, innovation has taken the form of new technological devices and therapeutics, which require patenting. The aim of this study is to identify major areas of innovation in the field of neurosurgery by evaluating patent records.

Methods

This study quantifies the number of patents the American Association of Neurological Surgeons (AANS) neurosurgeons hold across different subspecialties. The United States Patent and Trademark Office (USPTO) patent database was queried using the names of 7,293 AANS members who filed patents between 1976 and 2019.

Results

A total of 346 (4.7%) AANS neurosurgeons hold a total of 1,025 patents. The number of patents held by each neurosurgeon ranged from one to 109. The areas that patents were filed under include cellular and genetic science (40), drug delivery (45), image guidance (82), neuromodulation (52), pain (7), peripheral nerve stimulation (24), spine (398), surgical devices (148), trauma (16), tumor (78), vascular (67), and other (68). No patents were filed under pediatrics (0). The fields with the greatest number of filed patents are spine, instruments/devices, and image guidance.

Conclusion

Given the technical nature of the field of neurosurgery, instruments and devices that improve localization, visualization, targeting, and spinal reconstruction are often in demand. Furthermore, since the rates of spinal procedures and implants continue to increase, higher patenting may be motivated by the opportunity to develop new products that can result in royalty payments to neurosurgeons. The advent of new technologies undoubtedly continues to push the field of neurosurgery forward.

## Introduction

Over the last several decades, neurosurgeon-led innovations have resulted in the development of new therapeutics, imaging modalities, instrumentation, and devices that benefit patient treatment. Neurosurgeons have identified gaps in practice where their innovations have led to advancements in neurosurgery and unique collaborations with industry where patents have been issued. A previous review of neurosurgical innovation across 90 countries found that the top-performing patent categories were image-guidance, neurophysiology, and neuromodulation devices [1]. In the United States, the top three subspecialty areas in which patents are held include spine, tumor, and stereotaxy/image-guidance [2]. Spine typically garners significant interest, considering the high cost of implants and the routine use of pedicle screw/rod systems and interbody devices for spine reconstruction [3-4]. In the field of brain tumors, patents include novel methods of diagnosis/intraoperative detection, intracavitary drug treatments, and vaccine therapies [5-7]. The development of image-guided stereotactic radiosurgery (SRS) with the introduction of the Gamma Knife (Electa, Stockholm, Sweden) and CyberKnife (Accuray Incorporated, Sunnyvale, California) have provided targeting of cranial and spinal lesions with greater precision and accuracy than standard external beam radiation therapy with excellent long-term results [8-9]. Medtronic implantable electrodes (Medtronic plc, Dublin, Ireland) have been used in a range of disorders, including chronic pain, cerebral palsy, and epilepsy [10]. More recently, SynchroMed (Medtronic) was approved by the Food and Drug Administration (FDA) as an implantable device that dispenses medication intrathecally for the management of chronic pain [11]. These are just a few examples of innovation being used to advance the field of neurosurgery.

Neurosurgeons have filed patents in order to protect the intellectual property of their innovations and developments. According to the Patent and Trademark Office of the United States Department of Commerce, a patent is federal protection of the “right to exclude others from making, using, or selling” an invention or discovery for a 17-year-period [12]. Patents are a means of reimbursing innovators for their time, capacity, and financial resources by granting “ownership” of the idea [2]. Patenting an idea helps guard against competition and allots time for further development. Those against medical professionals patenting new technologies claim the practice is unethical because doing so delays the dissemination of technological advances to the medical community and patients since it takes approximately 35 months for the United States Patent and Trademark Office (USPTO) to process a patent application [2,13].

Prior to the passage of the Bayh-Dole Act of 1980, the government-maintained ownership of all federally funded patents and inventions at research institutions. As a result, institutions could not financially benefit from the patenting and licensing of technologies that were federally funded, preventing them from commercializing innovations, which led to many stalled developments and ventures. However, beginning in 1980, the Act allowed universities that received federal grant funding to maintain ownership of inventions, incentivizing them to patent and license ideas and share license income [14]. Innovators could now balance risks by retaining intellectual property and offset development costs. As expected, there was an upsurge in patent applications and licensing agreements following the passage of the Act [2,15]. From 1991-2000, new patent applications increased by 238%, licensing agreements by 161%, and royalties by more than 520% [2,13]. By 2016, 6,600 of the 151,000 (4%) USPTO-granted patents were assigned to U.S. academics, with a substantial proportion being in the field of medical technology [16]. Thus, patenting has become an alternate route to development, especially in drug discovery. While the National Institutes of Health (NIH) funds hypothesis-driven research, academic-industry partnerships provide funding for early-stage drug development prior to commercialization [17]. Previous studies have demonstrated greater success rates for pharmaceutical and medical technology developments as a result of collaborations between academic and industry partners [18-19].

A more common route of neurosurgery innovation involves a partnership with industry (e.g., Medtronic, DePuy, Zimmer, and others), in which commercial partners provide licensing agreements to incentivize neurosurgeons. In such partnerships, compensation practices and intellectual property ownership vary across institutions, which are defined strictly in the external activity agreements defined by the institution and commercial partner. These agreements highlight policies, reporting requirements, and rights that neurosurgeons are required to follow when engaging with a commercial partner. In some cases, device makers may own product patents and agree to compensate neurosurgeons in royalties for contribution to product development [2]. In order to remove financial conflicts, many such agreements stipulate that royalties are distributed only when other neurosurgeons utilize the technology or treatment. Following the Grassley-Kohl Physician Payment Sunshine Act of 2007, hospitals and academic institutions limited the payments physicians received for serving on company boards and banned speaker fees [20]. In 2013, in an effort to increase transparency between physicians and companies, the Physician Payments Sunshine Act mandated all U.S. drug and device manufacturers to report payments made to physicians annually [15]. The Open Payments Database (OPD), implemented under the Affordable Care Act and managed by the Centers for Medicare & Medicaid Services (CMS), discloses payments for travel, research, gifts, speaking fees, and royalties [21]. In 2015, the industry paid physicians a total of $2.4 billion [21]. When evaluating industry financial relationships, neurosurgery ranks highest, followed by orthopedics, otolaryngology, urology, and plastic surgery [22]. Yet, CMS records show that orthopedic surgeons received the highest maximum payments per physician ($38 million), followed by neurosurgery ($18 million) [21]. Of the total payments to neurosurgeons, 74% were for royalties and patent licenses [23]. With evidence of industry-physician relationships, there has been some concern over conflicts of interest.

With a rise in neurosurgical innovation and patenting, there is growing interest in understanding which areas in neurosurgery patents are filed under. The purpose of this study is to provide an updated analysis of patents held by AANS surgeons across selected subspecialties and conduct a more granular review of the USPTO database to identify the most commonly patented ideas within each subspecialty. As new technologies emerge on the market, reach news outlets, and become incorporated into operative practice, there may be misconceptions about where surgical innovation is prevalent. A detailed analysis through patent records will allow for a better representation of where neurosurgeons devote their intellectual and monetary resources to develop the subfield in which they work. The results of this study present areas that have been of greatest interest and reveal topics that can be further explored.

## Materials and methods

We searched patent records from the USPTO with the names of 7,293 AANS members, regardless of whether or not they were practicing. The USPTO Patent Full-Text and Image Database (PatFT) includes filed and granted patent records dating back to 1976.

AANS surgeon names were entered into the USPTO database in order to determine the number of patents they held. We verified that the patents were associated with the correct surgeon by ensuring that the state the surgeon practiced in matched the state listed for the patent holder. Patent titles were transferred into a spreadsheet for the 346 of the 7,293 surgeons that held patents. The list of AANS patents filed between 1976 to 2019 by the 346 members totaled 1,025 patents. Duplicate titles for the same patent were eliminated from our master list. Patent titles were further categorized into the fields of tumor, spine, vascular, trauma, imaging, pain, peripheral nerve, neuromodulation, pediatrics, surgical devices, therapeutics, cellular and genetic science, and other based on keywords. For instance, for spine, the keywords included: plate, screw, rod, fusion, vertebral, stabilizer, spinal, spine, fixation, implant, interbody, disc, and so on. Unique keywords were used for each field. For the patents that were not captured by this method, the authors of this paper systematically reviewed patent abstracts to determine the most appropriate category.

## Results

**Figure 1:**
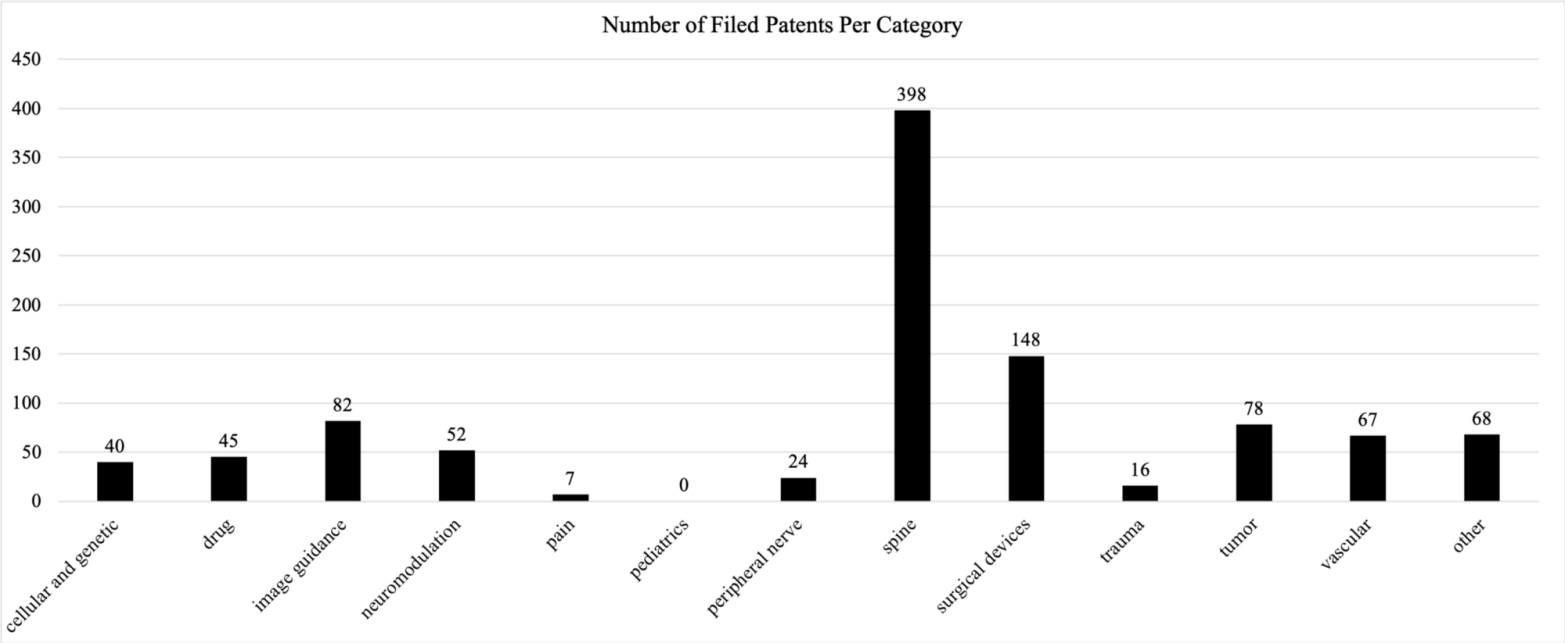
Number of patents filed by AANS members in each subfield American Association of Neurological Surgeons (AANS)

**Figure 2:**
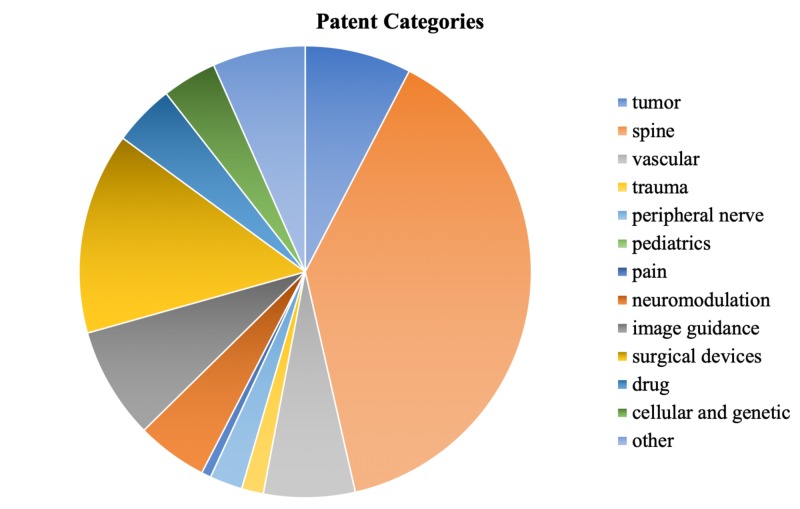
Percentage breakdown of patents in each subfield

Of the 7,293 AANS members in the registry, 346 (4.7%) of neurosurgeons held a total of 1,025 patents. The number of patents held by each of these neurosurgeons ranged from one to 109. Fields that patents were filed under included cellular and genetic science (40), drug delivery (45), image guidance (82), neuromodulation (52), pain (7), pediatrics (0), peripheral nerve stimulation (24), spine (398), surgical devices (148), trauma (16), tumor (78), vascular (67), and other (68) (Figure 1). The greatest number of patents were in the spine (39%), surgical devices (15%), and image guidance (8%) fields (Figure 2). Seven point four percent (7.4%) of patents did not fit into the previously stated categories and were therefore deemed “other.” Table 1 offers a breakdown of patent keywords that appeared within each subfield.

**Table 1:** Keywords that appeared in patent titles according to the subfield, listed in order of prevalence (not an exhaustive list) herpes simplex virus (HSV); Tumor necrosis factor-alpha (TNF-a); bis-chloroethylnitrosourea (BCNU); central nervous system (CNS); local binary pattern (LBP); brain-computer interface (BCI); electroencephalography (EEG)

Subfield	Keywords
Tumor	Vaccine, vector, HSV retrovirus, craniotomy, stem cells, TNF-a inhibitors, BCNU
Spine	Implant, plate, fusion, intervertebral disc, screw, spacer, interbody, screw, cage, expandable, minimally invasive, stabilizer, rod, spinal cord stimulation
Vascular	Aneurysm detection, clip, stroke, thrombolytic, blood pressure, plasminogen
Trauma	Subdural evacuation, assessing CNS integrity, head impact detection, sensor
Peripheral nerve	Stimulating DRG, stimulate neural tissues, blocking nerve conduction, modulation
Pediatrics	-
Pain	Rodent model, analgesic implant, methods for LBP
Neuromodulation	Deep brain stimulator, chemical modulation, magnetic modulation, implantable electrode, leads, BCI
Image guidance	Stereotaxy, radiosurgery, instrument guidance, frameless, 3-D beam localization, robotic, magnetic field digitizer, X-ray
Surgical devices	Medical device, apparatus, tissue biopsy, guide, needle, EEG, retractor, catheter, surgical instrument
Drug	Drug delivery, controlled release, dispensing pills, prodrug
Cellular and genetic science	Gene, expression, gene vector, analog, derivative, radionucleotide
Other	Surgical methods, compositions, fluid collection, 3D-model, radiation shielding, treating (other neurological conditions)

Spine (39%) consisted of the largest number of filed patents. The most common patents within this field were implants (20%), fusions (12%), plates (11%), discs (10%), and screws (9%). Most commonly, implants were intervertebral, expandable, and non-expandable. Fusion patents were often methods/techniques for fusion surgeries and devices/systems that assist in such procedures. Several patents for implants included plates and screws within the proposed system. Screws and plates were also mentioned in fixation systems.

The second most populous field was surgical devices (14%), which excluded patents with keywords for spine, image guidance, or neuromodulation. A significant portion of the devices was used to assist in surgeries (15%) such as a surgical knife, drape, mask, retractor, and so on. Common instruments included apparatuses and methods for tissue removal/retraction and biopsies (12%). Additional patents were instrument guides (8%) for a biopsy needle, surgical tool, or drill.

The most commonly filed patents under “image guidance” (8%) were for stereotactic radiosurgery (36%). These included stereotactic frames, fixation devices, instruments, and procedure methods. Related patents included three-dimensional beam localization apparatuses to determine the size, location, and features of lesions that could be used in stereotactic surgery.

Despite expectations of high levels of innovation in neuromodulation, this category comprised 5% of filed patents. This subfield included deep brain stimulation (DBS) as well as chemical, thermal, and magnetic neuromodulation. Sixty percent (60%) of patents in this subfield were related to DBS, including patents for electrodes, implantable devices, brain-computer interfaces, and treatment of neurological movement disorders.

A number of patents (7%) that did not fit into the previously determined categories were deemed as “other.” A majority of patents were for devices that could be used in the operating room that were not directly related to neurosurgery, such as fluid collection containers and techniques for illuminating the surgical space. Other patented ideas were for methods for reducing surgical error such as using three-dimensional models prior to operation and techniques to avoid wrong-site surgeries.

## Discussion

Many neurosurgeons today are clinicians, researchers, and innovators. Through years of experience with patients, concepts emerge to better address the cases they see. New ideas should be encouraged and cultivated, as the objective is to improve patient care. With new inventions, we also recognize the need to protect intellectual property through patents. Our analysis revealed that patents were filed in fields where there was greater demand, such as the need for better visualization intraoperatively and tools that improved technical dexterity. We also saw considerable innovation in fields where inventors could reap greater financial gains such as spine. These results follow predictions made by the United States Committee for Economic Development, which stated that the two greatest trends in university research were: (1) an increased number of industry-university collaborations and (2) financially profitable licensed ideas [24]. Surgical devices are more commonly filed with the USPTO (28%) than with the World Intellectual Property Organization (22%) or European Patent Office (8%) [25]. Japan and Israel are the leaders in medical device foreign origin patents [26].

The results of our study demonstrate that the field with the greatest number of patents and innovation is spine. There are several reasons why this may be the case, including increased demand for spinal procedures and greater profits to be made in patenting implants and other spinal devices. One study found that there has been a 62% increase in procedure volume for lumbar fusions from 2004 to 2015, with many more elective spinal fusions for degenerative conditions [27]. Furthermore, the microeconomy for spinal implants causes great variability in implant costs. Once innovations are patented and licensed to device manufacturers or vendors, they sign nondisclosure agreements with institutions that state negotiated prices will not be shared with others [28]. Thus, the cost of a single pedicle screw can range from $400-$1843, depending on manufacturer pricing and hospital payments [28]. This has led to a rise in cost-containment efforts that reduce costs and increase value in the setting of bundled payment programs.

Another driving factor for high levels of spine innovation may be the level of publicity that the field gained in recent years as neurosurgeons file lawsuits against device manufacturers for royalties that were not rendered. In a 2018 lawsuit, a spinal neurosurgeon won $112 million in royalties from Medtronic [29]. The field of spine generates a large amount of revenue for these companies. In 2018, Medtronic’s net spine sales approached $3 billion [30]. With more neurosurgeons patenting spinal instrumentation devices, they generate large sources of outside income solely from royalties.

The results of our analysis show that the second and third leading areas of innovation were “surgical devices” and “image guidance,” respectively. As neurosurgery requires great precision and accuracy, devices that improve the accuracy and trajectory of surgical tools are in demand. For example, brain biopsies need to be performed with caution in order to ensure that the needle is properly placed to avoid the risk of hemorrhage or other deficits. Not surprisingly, biopsy needles were among the most frequently patented instruments. Similarly, devices that fix the head in place to prevent motion are imperative during invasive neurosurgical procedures. The latter results are in concordance with a previously published study, which demonstrated that “image guidance devices” were among the top-performing clusters in neurosurgical technology [1]. Within this subfield, a majority of patents were filed under “stereotactic radiosurgery” (SRS). With a dramatic increase in the number of new radiosurgery technologies in the past three decades, SRS is considered a disruptive innovation [9].

Despite the widespread use of “neuromodulation” technologies, patents only comprised 5% of innovations held by AANS members in the USPTO database. A large driver of this subfield was “deep brain stimulation.” Patenting in this area may have weaned following the establishment of Medtronic’s Neurological Division, which largely produces DBS electrodes [2]. Following the initial concept, further patenting has refined the technology and expanded the neurological conditions for which the system could be used.

A limitation of this study is the possibility of human error when retrieving correct patent data from PatFT. Another limitation is that patents were placed into categories based on the researchers’ subjective assessment of an appropriate field. There may have been patents that could be categorized in more than one field, but they were placed according to what the researchers deemed most suitable. For instance, certain drug developments for brain tumors can either be categorized as “therapeutics” or “cellular and genetic science.” Similarly, a “vertebral distractor” is considered a “surgical instrument/device,” but since its primary use is for spinal fusion cases, it is categorized under “spine.” Further, the “other” category included patents that did not fit into any one category such as surgical approaches for treating other neurological conditions. In some cases, “other” patents were not directly related to neurosurgery, but since the objective of our study was to report patents filed by all AANS neurosurgeons, regardless of field relevance, they were not excluded. Lastly, while this study captures the level of patenting practices among neurosurgical surgeons, it does not assess earlier stages of ideation. Thus, an investigation into invention disclosures is warranted.

## Conclusions

The results of this study reveal trends in neurosurgical innovation, showing that the greatest number of patents are filed in the fields of spine, instruments/devices, and image guidance. The level of spinal innovation has increased in recent years, likely due to a rise in elective procedures and greater profits that could be made due to unregulated pricing for implants. Instruments and devices that assist in localization for neurosurgical procedures continue to be in demand due to the high level of precision required in the field. The rise in neurosurgery patents makes it clear that collaborations between physicians and industry will likely remain a continued trend. Ultimately, these partnerships and new technologies are important for advancing the field and improving patient treatment and outcomes.
